# Foramen magnum stenosis, cervicomedullary decompression, and growth in children with achondroplasia: a retrospective cohort study

**DOI:** 10.1186/s13023-026-04219-3

**Published:** 2026-01-29

**Authors:** Daniela Fava, Alessia Angelelli, Caterina Tedesco, Marta Panciroli, Silvia Bianchin, Daniela Teruzzi, Alessandra Scaravilli, Angelica Pisati, Isabella Barranca, Mariagrazia Scilipoti, Mariasavina Severino, Flavia Napoli, Nadia Gabriella Maiorano, Alessandro Naim, Sofia Negri, Giuseppa Patti, Angela Pistorio, Natascia Di Iorgi, Anna Elsa Maria Allegri, Mohamad Maghnie, The Skeletal Dysplasia Multidisciplinary Team (GM-MOC)

**Affiliations:** 1 Department of Neuroscience, Rehabilitation, Ophthalmology, Genetics, Maternal and Child Health, University of Genoa, Genoa, Italy; 2 Pediatric Endocrinology Unit, Department of Pediatrics, IRCCS Istituto Giannina Gaslini, University of Genoa, Via Gerolamo Gaslini 5, 16147 Genoa, Italy; 3 Department of Advanced Biomedical Sciences, University of Naples “Federico II”, Naples, Italy; 4 Neuroradiology Unit, IRCCS Istituto Giannina Gaslini, Genoa, Italy; 5 Scientific Directorate, Epidemiology and Biostatistics Unit, IRCCS Istituto Giannina Gaslini, Genoa, Italy

**Keywords:** Achondroplasia, Growth, Neurosurgery, Skeletal dysplasia, Foramen Magnum stenosis, Magnetic Resonance Imaging

## Abstract

**Background:**

Foramen magnum stenosis (FMS) is a serious complication in children with achondroplasia that may necessitate cervicomedullary decompression (CMD). It is unclear how FMS and CMD influence growth in these children. This study aimed to assess the effects of FMS and CMD on the growth of children with achondroplasia.

**Methods:**

Eighty-seven children (45 males, 42 females) with achondroplasia, aged 4 to 6 years, were evaluated. Height, weight, head circumference, and body mass index were expressed as standard deviation scores (SDS) according to Merker et al., while sitting height SDS was derived using Tanner’s methods. FMS was graded on magnetic resonance imaging using Fornarino’s score.

**Results:**

Fifty-two patients (26 males, 26 females) underwent CMD at a median age of 0.95 years (IQR 0.52;1.50). Of these, 28 (53.8%) were under one year old at the time of CMD, with a median age of 0.6 years (0.4;0.7). The remaining 24 children had CMD after their first year of life, with a median age of 1.6 years (1.3;2.8). The median age at anthropometric assessment was 5.16 years (4.74;5.50). Children who underwent CMD showed significantly lower median height SDS, particularly among males compared to females (p=0.026).

**Conclusions:**

Impaired growth in children with foramen magnum stenosis requiring cervicomedullary decompression may primarily reflect greater disease severity, while the potential contribution of surgery remains uncertain.

## Background

Achondroplasia (ACH) is the most prevalent form of skeletal dysplasia characterized by disproportionate short stature. The heterozygous point mutation p.Gly380Arg (c0.1138 G > A) in the fibroblast growth factor receptor 3 gene (FGFR3) accounts for 98–99% of cases [1]. This gain-of-function mutation in FGFR3 disrupts endochondral ossification, resulting in limb shortening and serious complications, such as foramen magnum stenosis (FMS) [2–5].

Dysregulated bone growth leads to thickening of the occipital rim, which assumes a more horizontal orientation than normal, along with overgrowth of the opisthion and early closure of the anterior and posterior inter-occipital synchondroses [3, 6]. FMS is often present at birth but tends to worsen within the first year of life [7]. The reduced sagittal and especially transverse diameters of the foramen magnum can compress the brainstem or upper cervical spinal cord. Clinically, this compression may manifest as central apneas, respiratory arrest, and even sudden death, with an estimated risk of 2 to 7.5% in infants with achondroplasia [2–4, 8]. Beyond acute life-threatening events, FMS can lead to neurological motor dysfunctions, potentially resulting in quadriparesis [9].

Magnetic resonance imaging (MRI)-based grading systems are now available to assess the severity of foramen magnum narrowing [7, 10]. However, the question of whether all infants with ACH should undergo brain MRI remains debated, given the high prevalence of smaller foramen magnum in this population [4, 9, 11–15]. In 2005, American guidelines reiterated a recommendation from 1995 advocating for brain imaging in all infants with achondroplasia [16]. By 2016, an international expert group suggested that brain MRI should only be performed if abnormalities are detected during physical examinations or polysomnography [16]. The 2021 International Consensus Statement on diagnosis, multidisciplinary management, and lifelong care of individuals with achondroplasia recommended that asymptomatic infants be considered for MRI scanning to evaluate the cervicomedullary junction and foramen magnum size during the first few months of life [17]. In 2023, the European Achondroplasia Forum provided updated guidelines for detecting FMS. These guidelines clarify existing recommendations regarding monitoring protocols, appropriate assessments, and actions to take when indicators of concern are identified in infants or in rare cases where symptoms arise after age 2 [4].

The presence of FMS on imaging studies does not always indicate clinically significant compression [7]. Sanders et al. pointed out the low sensitivity of this approach, noting that neurological symptoms other than hypotonia were identified in only 2 out of 27 patients with cervical spinal cord compression [9]. Similarly, central apneas were observed in just 2 of 23 patients with spinal cord compression who underwent polysomnography [9]. Cheung et al. found that neither neurological examinations nor sleep studies reliably predict the severity of FMS [7]. While there is a risk of overdiagnosis and unnecessary surgeries, the strong recommendation for brain imaging in infants with achondroplasia remains unchanged [4, 17]. Recently, the European Society of Pediatric Radiology and the European Society of Neuroradiology recommend conducting MRI upon the diagnosis of achondroplasia and whenever new neurological symptoms arise. They also proposed a standardized imaging protocol for MRI to guide clinical practice [18].

The rates of cervicomedullary decompression (CMD) vary widely, ranging from 4.6% to 43%, influenced by factors such as clinical practices and referral patterns [7, 9, 19–22]. Due to the absence of a consensus on the optimal timing for CMD, there is considerable variability in neurosurgical approaches across different centers. Notably, the median age at which CMD is performed has decreased significantly over the past four decades [23]. In the “Achondroplasia Natural History Study (CLARITY),” the median age for the first CMD was 1.3 years for males and 1.1 years for females, with 91% of children born after 2010 undergoing the procedure before the age of 2 [23].

A recent systematic review found that 21% of children experienced complications following CMD, including cerebrospinal fluid leaks, surgical site infections, respiratory issues, and a mortality rate of 3% [15]. Beyond the surgical risks, FMS and its management may also have broader clinical implications, including potential effects on growth. Children with achondroplasia exhibit a distinctive pattern of linear growth characterized by a markedly reduced height velocity from infancy onwards. Longitudinal studies have consistently shown that growth velocity is highest during the first year of life, approximately 12–15 cm/year, and then rapidly declines to about 3–4 cm/year during the preschool years, remaining low throughout childhood with minimal pubertal acceleration [24–27]. While the primary genetic defect is the main determinant of short stature in achondroplasia, additional factors such as neurological involvement, respiratory dysfunction, and reduced mobility may further modulate growth trajectories. Although several studies have reported associations between overall disease severity and impaired growth [25, 28–30], the possible contribution of FMS and its surgical management through CMD to growth outcomes remains largely unexplored.

The aim of this study was therefore to compare height and other anthropometric parameters measured at 4–6 years between children with achondroplasia who underwent cervicomedullary decompression for foramen magnum stenosis and those who did not.

## Patients and methods

This retrospective cohort study included prepubertal patients with genetically confirmed ACH, aged 4 to 6 years, who were followed at the Pediatric Endocrine Unit at IRCCS Istituto Giannina Gaslini, University of Genova, Italy, between January 2001 to November 2023. All patients were carried the same heterozygous point mutation in the FGFR3 gene, p.Gly380Arg (c0.1138 G > A); 86 had a de novo mutation, and one female had an affected parent. Anthropometric measurements were obtained from assessments performed at the 4–6-year visit, clinical history data were retrospectively collected from medical records to document events occurring before that visit. These data were used to compare children who underwent CMD in early childhood with those who did not. Children with uncontrolled systemic diseases known to interfere with growth were excluded.

## Data collection

### Anthropometric and clinicial data

Anthropometric data: Height (H in cm and SDS), growth velocity (GV in cm/year and SDS), weight (W in kg and SDS), head circumference (HC SDS), sitting height (SH SDS), body mass index (BMI SDS). Standard deviation scores (SDS) were calculated using Merker’s reference charts for ACH [31], except for GV and SH, calculated according to Tanner [32]. Height and sitting height were measured to the nearest 0.1 cm, while weight was measured to the nearest 0.1 kg using a calibrated Harpenden stadiometer and an electronic scale.

The main analysis was based on anthropometric measurements obtained during the clinical evaluation performed between 4 and 6 years of age. When available, additional anthropometric data (H SDS, W SDS, HC SDS, BMI SDS) from earlier assessments during the first two years of life were also retrieved. In patients who underwent CMD, only data from a preoperative visit were considered, whereas for non-operated patients, measurements from their first evaluation at our center were used, if performed before the 4–6-year visit.

Clinical variables comprised sex, age at evaluation, pubertal Tanner stage, ethnicity, age at CMD, presence of comorbidities and postoperative complications. Recorded complications included myelopathy, hypotonia, quadriparesis, hydrocephalus requiring ventriculoperitoneal shunting, and need for repeated CMD. Additional conditions, including celiac disease and orthopedic or respiratory comorbidities, were recorded (genu varum, lumbar kyphosis, need for continuous positive airway pressure [CPAP], and adenoidectomy/tonsillectomy). Results of somatosensory evoked potentials (SEP), used to assess spinal cord function, and of sleep studies performed to detect central or obstructive apneas were also retrospectively retrieved from medical records.

### Neuroradiological data

Cranio-spinal MRI studies were acquired from January 2001 to August 2020 with different scanners, including 1.5T scanners (Achieva; Philips Medical Systems, Best, the Netherlands), with the same protocols including among the others 3 mm-thick T2-weighted images acquired in the sagittal plane in the neutral position.

The Fornarino’s score could be calculated in 48 out of 52 cases, as MRI images were not available for the remaining four patients, for whom only the radiological report was accessible.

MR images were evaluated in consensus by two pediatric neuroradiologists (AS and MS) with 4 and 15 years of experience respectively, blinded to the subject’s clinical data. The cervical stenosis was graded according to a semi-quantitative score at the level of the foramen magnum/C1 [10]. Briefly, grade 0 indicated absence of signs of focal subarachnoid spaces reduction. Grade 1, 2 and 3 indicated mild, moderate and severe FMS, respectively. Grade 3 was defined by the presence of spinal cord compression without T2 signal alterations of the spinal cord. The presence of cervical T2 hyperintense intra-medullary lesion combined with absent, mild and moderate FMS defined a grade 4A, while the same lesion associated with a severe FMS defined a grade 4B. The MRI scan used for Fornarino’s score evaluation corresponded to the preoperative study in children who underwent CMD, and to the MRI performed prior to the clinical visit at which anthropometric data were collected in non-operated patients. MRI evaluation included assessment of posterior cerebrospinal fluid flow, and in selected cases, a complementary CT scan of the cranio-cervical junction was performed.

### Statistical analysis

Descriptive statistics were performed; categorical variables were reported in terms of absolute frequencies and percentages. Quantitative variables were reported in terms of median values and first and third quartiles (1^st^–3^rd^ q), as the data distribution was skewed. Normality of the distributions was tested by means of the Shapiro-Wilk test. Comparison of frequencies was done using the Chi-square test or the Fisher’s exact test (in case of expected frequencies less than 5). Comparison of quantitative variables in 2 different categories of patients was made by the non-parametric Mann-Whitney U test.

Multiple regression model was performed to evaluate the relationship between height reached at 4–6 years of age and some exposure variables: CMD, sex, Fornarino’s score and age at CMD < 1 years; regression coefficients adjusted for the variables included in the model (β_adj_) and their Standard Errors were reported in the tables. Another multiple regression model was performed using height SDS as an outcome variable. All the statistical tests were two-sided and a *p* value < 0.05 was considered statistically significant. The software “Stata” (release 17.0, College Station, TX, USA) was used for all the univariate and bivariate analyses and Statistica (release 9.1, StatSoft Corporation, Tulsa, OK, USA) was used for the multivariable regression models.

## Results

**Fig. 1:**
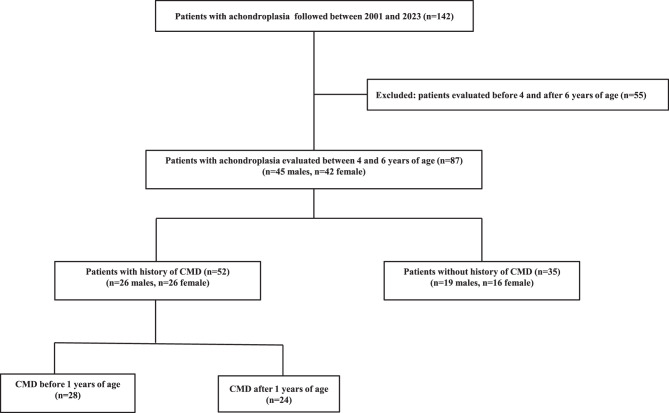
Flowchart for selection of patients based on the adopted criteria for the study

During the study period (January 2001–November 2023), a total of 142 patients with ACH were followed at the Pediatric Endocrine Unit. Of these, 87 prepubertal children (45 males, 42 females) were evaluated between the ages of 4 and 6 years, with a median age of 5.16 years (IQR 4.74; 5.50) (Fig. 1). The median values of anthropometric measurements for the entire cohort were within the normal range at the time of evaluation. Apart from two boys from South American families, all children in the cohort were of European origin.

Among the participants, 52 patients (59.8%)—26 males and 26 females—underwent CMD at a median age of 0.95 years (IQR 0.52; 1.50). No significant differences were observed between children who underwent CMD and those who did not regarding age at visit, W SDS, BMI SDS, HC SDS, SH SDS, and the SH-H ratio (Table 1). However, H SDS was significantly lower in children who underwent CMD (*p* = 0.03).

**Table 1:** Age, anthropometrics and Fornarino’s score for foramen magnum stenosis in patients with and without CMD

	CMD [*n* = 52]	No CMD [*n* = 35]	P value
	***Median (1***^*st*^*− 3*^*rd*^*q)*	***Median (1***^*st*^*− 3*^*rd*^*q)*	
Age at visit (years)	5.13 (4.68; 5.44)	5.22 (4.75; 5.68)	0.16#
Height SDS	−0.69 (−1.35; 0.28)	0.05 (−0.65; 0.66)	0.030#
Weight SDS	−0.54 (−1.14; 0.37)	−0.12 (−0.78; 0.61)	0.16#
BMI SDS	0.005 (−0.79; 0.36)	−0.275 (−0.74; 0.52)	0.84#
Head circumference SDS	−0.6 (−1.15; 0.35)	−0.4 (−1.3; 0.15)	1.00#
Sitting height SDS	−1.6 (−2.2; −0.8) [*n* = 31]	−1.2 (−2.1; −0.6) [*n* = 19]	0.42#
Sitting height/Height Ratio	0.68 (0.67; 0.69)	0.68 (0.66; 0.70)	0.98#
Fornarino’s Score*	3 (1; 4B)	0 (0; 1)	< 0.0001#

CMD: cervico-medullary decompression. ^*#*^P: Mann-Whitney U test. * Reference 10

The Fornarino’s score was significantly higher in patients who underwent CMD compared to those who did not (*p* < 0.001). Brain MRI revealed mild-moderate FMS (Fornarino’s grades 1 and 2) in 17 patients (10 males and 7 females) who underwent CMD, and in 13 patients who did not undergo CMD (6 males and 7 females). Significant stenosis or cervical intramedullary lesions (grades 3, 4A, and 4B) were identified in 31 operated patients (14 males and 17 females) and in 4 non-operated patients (2 males and 2 females). Fornarino’s score data were missing for 4 operated patients.

In males, both H and H SDS were significantly lower in those who underwent CMD compared to non-operated males (*p* = 0.002 and *p* = 0.018, respectively), while no significant differences were found in females. Among children who underwent CMD, height SDS were significantly lower in boys than in girls (*p* = 0.026), while among non-operated children, no significant difference in height SDS was found between sexes (Table 2).

**Table 2:** Clinical variables and anthropometric parameters of 87 patients analyzed based on gender (males vs females) and CMD (yes vs no)

		Males [*n* = 45]	Females [*n* = 42]	P value
		***Median (1***^*st*^*− 3*^*rd*^*q)*	***Median (1***^*st*^*− 3*^*rd*^*q)*	
CMD: yes	Age at visit (years)	5.14 (4.74; 5.48)[*n* = 26]	5.11 (4.62; 5.4)[*n* = 26]	0.81#
no		5.39 (5.01; 5.69)[*n* = 19]	4.97 (4.7; 5.37)[*n* = 16]	0.10#
		0.06^*#*^	0.97^*#*^	
CMD: yes	Age at CMD (years)	1.29 (0.62; 2.51)	0.77 (0.51; 1.23)	0.06#
CMD: yes	Height (cm)	84.4 (82.7; 87)[*n* = 26]	86.05 (82.1; 87.5)[*n* = 26]	0.75#
no		88.9 (86.2; 92.2)[*n* = 19]	85.85 (83.7; 88.4)[*n* = 16]	0.013#
		0.002^*#*^	0.70^*#*^	
CMD: yes	Height SDS	−1.12 (−1.45; −0.1)[*n* = 26]	0.17 (−0.65; 0.67)[*n* = 26]	0.026#
no		−0.23 (−0.71; 0.3)[*n* = 19]	−0.09 (−0.63; 0.63)[*n* = 16]	0.73#
		0.018^*#*^	0.57^*#*^	
CMD: yes	Growth velocity (cm)	3.9 (3.1; 4.5) [*n* = 15]	4.5 (3.25; 5.1) [*n* = 20]	0.35#
no		4.5 (2; 5.4) [*n* = 9]	3.5 (3.15; 4.8) [*n* = 8]	0.69#
		0.61^*#*^	0.43^*#*^	
CMD: yes	Growth velocity SDS	−2.8 (−3.3; −1.9) [*n* = 15]	−1.95 (−3.2; −1.65) [*n* = 20]	0.23#
no		−1.9 (−4.4; −1) [*n* = 9]	−2.8 (−3.3; −2) [*n* = 8]	0.53#
		0.53^*#*^	0.30^*#*^	
CMD: yes	Weight (Kg)	14.4 (13.5; 15.7) [*n* = 26]	14.65 (13.6; 15.9) [*n* = 26]	0.65#
no		15.5 (14.2; 17.2) [*n* = 18]	15.15 (13.7; 16.55) [*n* = 16]	0.53#
		0.09^*#*^	0.53^*#*^	
CMD: yes	Weight SDS	−0.85 (−1.51; −0.38)[*n* = 26]	−0.03 (−0.65; 0.47)[*n* = 26]	0.019#
no		−0.41 (−1.07; 0.36)[*n* = 18]	0.21 (−0.27; 0.64)[*n* = 16]	0.17#
		0.19^*#*^	0.41^*#*^	
CMD: yes	BMI SDS	0.03 (−0.82; 0.23)	−0.001 (−0.75; 0.54)	0.39#
no		−0.47 (−0.78; −0.05)	0.22 (−0.39; 0.79)	0.08#
		0.36^*#*^	0.57^*#*^	
CMD: yes	Head circumference SDS	−0.68 (−1.15; 0.2)	−0.35 (−0.85; 0.4)	0.39#
no		−0.40 (−1.20; 0.15)	−0.35 (−1.30; 0.15)	0.94#
		0.99^*#*^	0.81^*#*^	
CMD: yes	Sitting height SDS	−1.85 (−2.4; −1.3)[*n* = 14]	−1.5 (−1.9; −0.8)[*n* = 17]	0.23#
no		−1.65 (−2.4; −0.6)[*n* = 10]	−0.9 (−1.3; −0.6)[*n* = 9]	0.29#
		0.83^*#*^	0.32^*#*^	
CMD: yes	Sitting Height/Height Ratio	0.68 (0.67; 0.69) [*n* = 14]	0.68 (0.67; 0.69) [*n* = 17]	0.52#
no		0.67 (0.66; 0.7) [*n* = 10]	0.68 (0.68; 0.69) [*n* = 9]	0.50#
		0.34^*#*^	0.37^*#*^	
CMD: yes	Fornarino’s Score	3 (1; 5) [*n* = 24]	4 (2; 5) [*n* = 24]	0.33#
no		0 (0; 1) [*n* = 19]	1 (0; 1) [*n* = 16]	0.67#
		< 0.0001^*#*^	< 0.0001^*#*^	

^*#*^ P: Mann-Whitney U test

Among the operated patients, W SDS was significantly lower in males compared to females (*p* = 0.019). However, no significant differences in W SDS were found between operated and non-operated males or between operated and non-operated females.

**Fig. 2:**
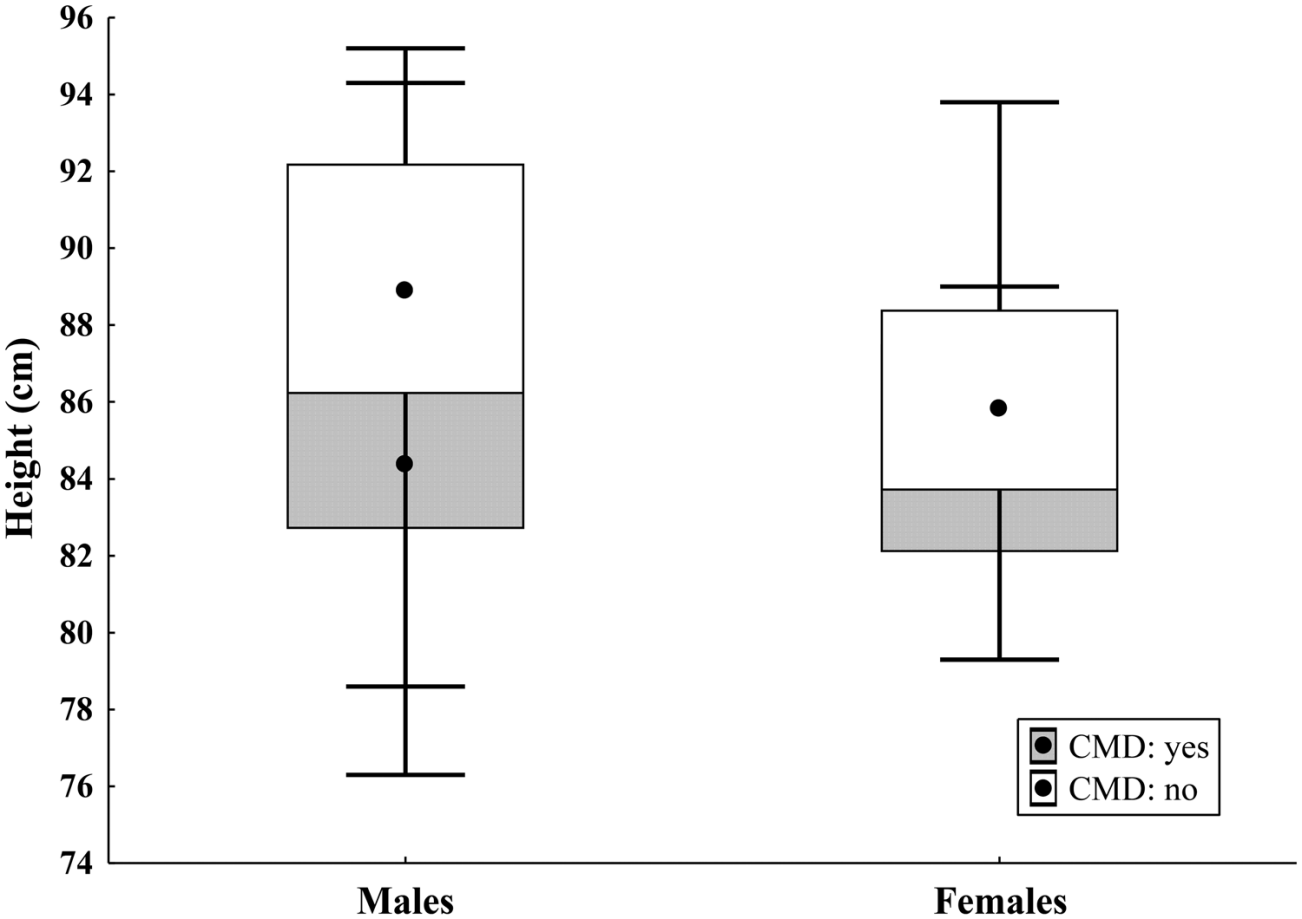
Correlation between cervicomedullary decompression surgery and height at 5 years, split by sex; simple bivariate analysis

We evaluated the correlation between H, H SDS, and key patient characteristics, including sex, CMD surgery, age at CMD, and Fornarino’s score, using multiple regression models. A strong inverse correlation was found between H and CMD surgery (β_Adj_ = −2.19; *p* = 0.012), correcting for the effect of sex (Table 3, at top), as illustrated in Fig. 2, which presents a simple bivariate analysis.

**Table 3:** Multiple regression model: outcome: height (cm)

Exposure variables	_**βAdj**_	SE (β)	P
**All patients, N = 87**			
Intercept	87.75	0.76	
Sex (Male: 0; Female: 1)	− 1.04	0.84	0.22
CMD (No: 0; Yes: 1)	− 2.19	0.86	0.012
**Only CMD patients, N = 52**			
Intercept	86.71	1.74	
Sex (Male: 0; Female: 1)	0.78	1.10	0.48
CMD < 1 year (No: 0; Yes: 1)	− 1.34	1.10	0.23

βAdj: Regression coefficient of the exposures adjusted for the role of the second exposure factor; SE (β): Standard Error of β

When analyzing only the CMD patients, the multiple regression model showed no significant correlation between H and age at CMD (less than 1 year), even after adjusting for sex (Table 3, at bottom). The negative correlation with CMD remained significant when H SDS was considered as the outcome variable (Table 4) (β_Adj_ = −0.47; *p* = 0.027).

**Table 4:** Multiple regression model: outcome: height-SDS

Exposure variables	_**βAdj**_	SE (β)	P
**All patients, N = 87**			
Intercept	− 0.13	0.19	
Sex (Male: 0; Female: 1)	0.23	0.20	0.26
CMD (No: 0; Yes: 1)	− 0.47	0.21	0.027

βAdj: Regression coefficient of the exposures adjusted for the role of the second exposure factor; SE (β): Standard Error of β

Twenty-eight of the 52 patients who underwent CMD were younger than one year at the time of surgery (Table 5). No differences in anthropometric parameters were found between these two subgroups. The Fornarino’s score was significantly higher in children who underwent surgery before the age of one year compared to those treated later (*p* = 0.049).

**Table 5:** Clinical variables and anthropometric parameters of 52 patients who underwent CMD categorized by age at the time of surgery ( < 12 months vs. ≥ 12 months)

	< 12 months at CMD [*n* = 28]	≥ 12 months at CMD [*n* = 24]	P value
	***Median (1***^*st*^*− 3*^*rd*^*q)*	***Median (1***^*st*^*− 3*^*rd*^*q)*	
Age (years)	5.1 (4.5; 5.3)	5.3 (4.9; 5.5)	0.06#
Age at surgery (years)	0.6 (0.4; 0.7)	1.6 (1.3; 2.8)	< 0.0001#
Height SDS	−0.26 (−1.23; 0.27)	−0.96 (−1.37; 0.3)	0.40#
Weight SDS	−0.38 (−1.04; 0.4)	−0.76 (−1.47; 0.09)	0.22#
BMI SDS	0.08 (−0.6; 0.57)	0 (−0.99; 0.23)	0.32#
Head circumference SDS	−0.6 (−0.9; 0.4) [*n* = 25]	−0.63 (−1.3; 0.35)	0.56#
Sitting Height/Height Ratio	0.68 (0.67; 0.69) [*n* = 15]	0.68 (0.67; 0.7) [*n* = 16]	0.77#
Fornarino’s Score	4 (3; 4B) [*n* = 27]	2 (1; 4B) [*n* = 21]	0.049#

^*#*^ P: Mann-Whitney U test. CMD: cervico-medullary decompression

Of the 87 patients, 49 (23 males and 26 females) had an earlier visit within the first two years of life; 31 of these (16 males and 15 females) were operated patients with a pre-CMD visit. (Table 6). For these patients, the median time interval between the first evaluation and neurosurgery was 0.15 years (IQR 0.09; 0.34). The age at first visit was significantly younger for patients undergoing CMD (*p* = 0.03), particularly among females (*p* = 0.005) (Table 7). No significant differences in anthropometric parameters were observed between patients who underwent CMD and those who did not, including when stratified by sex (Table 7) or by age at surgery ( < 1 year vs > 1 year; Table 8).

**Table 6:** Early anthropometric parameters in a subgroup of patients (*n* = 49) at the time of first referral

	CMD [*n* = 31]	No CMD [*n* = 18]	P value
	***Median (1***^*st*^*− 3*^*rd*^*q)*	***Median (1***^*st*^*− 3*^*rd*^*q)*	
Age (years)	0.65 (0.42; 1.41)[*n* = 31]	1.24 (0.93; 1.91)[*n* = 18]	0.033#
Height SDS	−0.54 (−1.44; 0.19)[*n* = 31]	−0.14 (−0.67; 0.54)[*n* = 18]	0.22#
Weight SDS	−0.70 (−1.51; 0.00)[*n* = 29]	−0.26 (−0.91; 0.49)[*n* = 17]	0.14#
BMI SDS	−0.51 (−0.81; −0.12)[*n* = 29]	−0.04 (−0.70; 0.71)[*n* = 17]	0.12#
Head circumference SDS	−0.33 (−1.31; −0.06)[*n* = 29]	−0.75 (−0.94; 0.67)[*n* = 13]	0.88#

CMD: cervico-medullary decompression #P: Mann-Whitney U test

**Table 7:** Clinical variables and anthropometric parameters of 49 patients at first visit analyzed based on gender (males vs. females) and CMD (yes vs. no)

		Males [*n* = 23]	Females [*n* = 26]	p value
		***Median (1***^*st*^*− 3*^*rd*^*q)*	***Median (1***^*st*^*− 3*^*rd*^*q)*	
CMD: yes	Age	1.2 (0.47; 2.8) [*n* = 16]	0.5 (0.34; 1.1)[*n* = 15]	0.049#
No		1.5 (0.97; 2.2) [*n* = 7]	1.2 (0.7; 1.6)[*n* = 11]	0.37#
		0.62^*#*^	0.005^*#*^	
CMD: yes	Height SDS	−0.40 (−1.51; 0.74) [*n* = 16]	−0.54 (−1.44; −0.04)[*n* = 15]	0.40#
No		−0.18 (−0.77; 0.96) [*n* = 7]	−0.10 (−0.67; 0.54)[*n* = 11]	0.77#
		0.67^*#*^	0.18^*#*^	
CMD: yes	Weight SDS	−0.53 (−1.31; 0.19) [*n* = 16]	−0.79 (−1.8; 0) [*n* = 13]	0.37#
No		−0.24 (−0.92; 0.91) [*n* = 7]	−0.32 (−0.91; 0.49) [*n* = 10]	0.67#
		0.42^*#*^	0.17^*#*^	
CMD: yes	BMI SDS	−0.46 (−0.83; −0.07) [*n* = 16]	−0.53 (−0.66; −0.12) [*n* = 13]	0.92#
No		0.15 (−0.84; 0.71) [*n* = 7]	−0.13 (−0.7; 1.17) [*n* = 10]	0.74#
		0.41^*#*^	0.23^*#*^	
CMD: yes	Cranic circumference SDS	−0.33 (−1.67; −0.05) [*n* = 15]	−0.34 (−1.14; −0.06) [*n* = 14]	0.62#
No		−0.64 (−0.94; 0.67) [*n* = 6]	−0.8 (−1.37; 0.78) [*n* = 7]	1.00#
		0.61^*#*^	0.87^*#*^	
CMD: yes	Interval between first visit and surgery	0.1 (0.06; 0.3)[*n* = 16]	0.2 (0.1; 0.5)[*n* = 15]	0.26#

CMD: cervico-medullary decompression #P: Mann-Whitney U test

**Table 8:** Clinical variables and anthropometric parameters in 31 patients at first visit prior to CMD analyzed based on age category at surgery ( < 12 months vs. ≥ 12 months)

	< 12 months at surgery [*n* = 16]	≥ 12 months at surgery [*n* = 15]	p value
	***Median (1***^*st*^*− 3*^*rd*^*q)*	***Median (1***^*st*^*− 3*^*rd*^*q)*	
Age (years)	0.4 (0.2; 0.5)[*n* = 16]	1.4 (1.2; 3.1)[*n* = 15]	< 0.0001#
Height SDS	−0.14 (−1.79; 0.52)[*n* = 16]	−0.57 (−1.13; 0.05) [*n* = 15]	0.91#
Weight SDS	−0.71 (−1.5; 0.31)[*n* = 14]	−0.7 (−1.64; 0) [*n* = 15]	0.72#
BMI SDS	−0.54 (−0.81; −0.03)[*n* = 14]	−0.40 (−1.46; −0.12) [*n* = 15]	1.00#
Head circumference SDS	−0.33 (−1.67; 0.75)[*n* = 15]	−0.34 (−1.21; −0.21) [*n* = 14]	0.72#

CMD: cervico-medullary decompression #P: Mann-Whitney U test

In our cohort, among the 31 operated patients with both preoperative and 4–6-year evaluations available, we found no significant differences in anthropometric measures except for BMI SDS, which was significantly lower at the preoperative visit (*p* = 0.009) (Table 9).

**Table 9:** Anthropometric parameters in operated patients who underwent CMD at the preoperative visit and at 4–6 years of age

	Preoperative visit [*n* = 31]	Visit at 4–6 years of age [*n* = 31]	P value
	***Median (1***^*st*^*− 3*^*rd*^*q)*	***Median (1***^*st*^*− 3*^*rd*^*q)*	
Age	0.7 (0.4 -1.4)	5.1 (4.6 - 5.4)	
H_SDS	−0.5 (−1.4 - 0.2)	−0.7 (−1.4 - 0.3)	0.65#
Weight SDS	−0.7 (−1.5 - 0.0)	−0.5 (−0.9 - 0.4)	0.18#
BMI SDS	−0.5 (−0.8 - −0.1)	0.1 (−0.4 - 0.3)	0.009#
CC SDS	−0.3 (−1.3 - −0.1)	−0.7 (−1.1 - −0.2)	0.63#

CMD: cervico-medullary decompression #P: Wilcoxon test

Three out of 52 CMD patients (1 male, 2 females), all with a Fornarino’s score of 4B, experienced severe myelopathy after surgery; postoperative complications included marked hypotonia or quadriparesis. Only one of these females had short stature at the time of evaluation (H SDS −2.32) and a weight deficit (W SDS −3.51; BMI SDS −1.89). Following CMD, she developed hydrocephalus and required a ventriculoperitoneal shunt. The male patient was the only one in the cohort to undergo a second CMD two years later due to recurrence of upper cord compression. No further evidence of developmental motor delay was observed in the remaining cohort. Two patients (1 male, 1 female) in the entire cohort were diagnosed with celiac disease. At the time of diagnosis, at ages 3.2 and 2.9 years, their anthropometric parameters were within the normal range. After starting a gluten-free diet, their IgA anti–tissue transglutaminase antibodies became undetectable within a few months. Excluding these five patients from the analysis did not significantly alter the results of the multiple regression model, which confirmed CMD as the only statistically significant variable (β_Adj_ = −1.83; SE (β) = 0.87; *p* = 0.037).

Among non-operated patients, three children with a Fornarino’s score of 4A and one male with a score of 4B had height SDS values ranging from −0.86 to +0.74 SDS at the 4–6-year evaluation. The boy with a score of 4B had a height SDS of −0.64 and presented with genu varum, whose severity could not be quantified from available clinical records, and obstructive sleep apneas documented by polysomnography, despite having previously undergone adenoidectomy. Due to sleep-disordered breathing, nocturnal CPAP therapy had been initiated approximately one year before the 4–6-year visit, with complete resolution of symptoms.

There were no significant differences in the incidence of limb and spinal deformities between patients who underwent CMD and those who did not (Table 10), even when considering sex differences. Additionally, no differences were found in the need for non-invasive ventilatory support, whether comparing individuals who underwent CMD before the age of 1 year or those who had surgery later. The percentage of subjects who underwent adenoidectomy was similar between operated and non-operated patients.

**Table 10:** Orthopedic and ENT comorbidities in patients with and without CMD

		CMD [*n* = 52]	No CMD [*n* = 35]	P value
Limbs deformity:	varism	25/46 (54.3%)	13/29 (44.8%)	0.77§
	valgus	7/46 (15.2%)	5/29 (17.2%)	
	none	14/46 (30.4%)	11/29 (37.9%)	
Dorso-lumbar kyphosis:	yes	11/44 (25%)	4/30 (13.3%)	0.22§
	no	33/44 (75%)	26/30 (86.7%)	
Hyperlordosis:	yes	26/44 (59.1%)	17/29 (58.6%)	0.97§
	no	18/44 (40.9%)	12/29 (41.4%)	
Non-invasive ventilation:	yes	7/46 (15.2%)	4/29 (13.8%)	1.00§§
	no	39/46 (84.8%)	25/29 (86.2%)	
Adeno-tonsillectomy:	yes	10/46 (21.7%)	6/29 (20.7%)	0.91§
	no	36/46 (78.3%)	23/29 (79.3%)	

CMD: cervico-medullary decompression §P: Chi-square test; §§ P: Fisher’s Exact test. All percentages reported in the table are column percentages

## Discussion

In this study, at 4–6 years of age, children with achondroplasia who underwent CMD for foramen magnum stenosis had lower height SDS compared to non-operated peers. To our knowledge, this is the first investigation linking CMD with height and weight outcomes in a large cohort of children with achondroplasia. Short stature is a defining characteristic of achondroplasia, with early growth deviations evident from the first months of life [23, 26, 31]. Specific growth curves have revealed slight variations among populations, likely reflecting ethnic or environmental influences, although average height and weight are broadly similar between 5 and 10 years [25].

Consistent with previous findings [31], non-operated boys in our cohort were taller than girls in absolute terms, though no significant sex difference was seen in height SDS at this age. Adult height differences between males and females vary from 7 to 12 cm across studies [27, 33, 34], with most sex-related height discrepancies emerging during adolescence. In our cohort, growth retardation appeared more pronounced among operated males, suggesting that underlying disease severity or factors related to early neurosurgical intervention may differentially affect male growth trajectories. This pattern was independent of age at surgery and not evident before CMD. Early-visit measurements may have been influenced by missing data and referral bias, as initial evaluations were available for only 56.3% of the cohort and 59.6% of operated patients. Operated children were younger at their first assessment, particularly females, reflecting our institution’s role as a tertiary referral center where CMD is performed shortly after initial evaluation.

Multiple regression models indicated that CMD predicted height SDS after correcting for sex, without a significant correlation with age at surgery < 1 year. Interestingly, sitting height SDS was generally normal, suggesting that the additional growth impairment primarily affects the lower extremities, consistent with the FGFR3 gain-of-function mutation inhibiting appendicular skeletal growth [35, 36]. In our cohort, median sitting height SDS aligned with healthy children [35, 37]. Fifteen patients had sitting height SDS <–2, with a higher proportion of operated females (80%) affected, whereas the percentage of males below −2 SDS was equal in operated and non-operated groups. Overall, no significant differences in sitting height SDS were found between sexes or between operated and non-operated children.

Weight SDS was lower in operated males compared to operated females, but no significant differences were seen between operated and non-operated groups or across sexes, contrasting with European growth charts where males typically maintain higher weight percentiles [23, 38]. Indeed, these differences in height and weight were not associated with orthopedic or respiratory complications, which occurred with similar frequency in both groups. Genu varum and lumbar kyphosis were common, slightly more prevalent in operated patients, but the severity of leg bowing could not be consistently documented. Respiratory complications, including the need for non-invasive ventilation, were comparable to prior reports [39], and adenotonsillectomy was less frequent than in other series [40–42].

Our study revealed a higher rate of CMD (compared to 4.5–42.2% reported in the literature) [22, 23, 43, 44], reflecting referral patterns to a tertiary center for multidisciplinary management. The reduced height in the CMD group likely reflects early disease severity or intrinsic vulnerability rather than a direct surgical effect. Children undergoing CMD often present with more severe neurological or respiratory involvement [41, 45], which may indirectly affect nutrition, activity, and growth. Within-patient analyses showed no change in height SDS from the early visit to the 4–6-year evaluation, supporting the notion that reduced growth reflects pre-existing vulnerability rather than surgery. Although a few isolated cases developed severe postoperative myelopathy, these did not result in persistent motor delays across the operated subgroup.

The relationship between early surgery and growth is complex. While some interventions for specific medical conditions may improve overall health and indirectly support growth [46–48], direct effects on stature are less clear, particularly when confounding factors such as low birth weight or congenital anomalies exist [49–51]. In our cohort, CMD was occasionally performed despite mild-to-moderate radiological stenosis, guided by abnormal somatosensory evoked potentials, reduced posterior CSF flow, central apneas, or progressive MRI findings. Surgical decisions were based on comprehensive multidisciplinary evaluation involving neurosurgery, neuroradiology, pediatrics, and neurophysiology [4, 10, 22, 52].

Although cervical spinal cord compression may resolve spontaneously with growth [22], CMD is essential to prevent life-threatening or irreversible neurological damage [15, 21]. Given the variable phenotypic expression of achondroplasia, predicting individual risks for FMS or growth impairment remains challenging. The potential influence of CMD on early growth trajectories warrants further exploration in larger cohorts with careful longitudinal monitoring. European consensus emphasizes early diagnosis, referral, and multidisciplinary management in the first two years of life to identify children with significant stenosis and associated growth impairments, enabling timely intervention [53–55].

## Conclusions

In our cohort, children with achondroplasia who underwent CMD for foramen magnum stenosis exhibited greater growth impairment in early childhood compared with non-operated peers. This likely reflects higher intrinsic disease severity and early-life health challenges, including subtle neurological or respiratory involvement, rather than a direct surgical effect, as sitting height SDS was largely preserved and within-patient analyses showed stable height trajectories from initial evaluation to 4–6 years. Males appeared particularly affected, suggesting that sex-related factors and early vulnerability may further influence growth. Height differences were independent of orthopedic or respiratory complications, which occurred at similar rates in both groups. These findings highlight the importance of early diagnosis, comprehensive multidisciplinary assessment, and close monitoring of growth in children with achondroplasia, and underscore the need for further studies to clarify the interactions between disease burden, timing of CMD, and long-term growth outcomes.

## Data Availability

The datasets used and/or analyzed during the current study are available from the corresponding author on reasonable request

## Abbreviations

ACH: Achondroplasia
BMI-SDS: Body mass index standard deviation scores
CMD: Cervicomedullary decompression
FGFR3: Fibroblast growth factor receptor 3 gene
FMS: Foramen magnum stenosis
GV: Growth velocity
H: Height
HC-SDS: Head circumference standard deviation scores
H-SDS: Height standard deviation scores
MRI: Magnetic resonance imaging
SH-H ratio: Sitting height – height ratio
SH-SDS: Sitting height standard deviation scores
CPAP: Continuous positive airway pressure
SEP: Somatosensory evoked potentials
W: Weight
W-SDS: Weight standard deviation scores
